# Value of inflammation and nutrition markers in predicting the failure of prosthesis removal and antibiotic bone cement spacer implantation for PJI treatment

**DOI:** 10.3389/fcimb.2025.1610156

**Published:** 2025-12-19

**Authors:** Jincheng Huang, Songtao Han, Xuguang Cheng, Meng Zhang, Zongyan Gao, Xiao Chen, Dapeng Wu, Tao Liu, Yi Jin

**Affiliations:** 1 Department of Orthopedics, Henan Provincial People’s Hospital, Henan University People’s Hospital, Zhengzhou University People’s Hospital, Zhengzhou, Henan, China; 2 Department of Orthopedics, Third Affiliated Hospital of Henan University of Traditional Chinese Medicine, Zhengzhou, Henan, China; 3 Department of Orthopedics, Hebi People’s Hospital, Hebi, Henan, China; 4 Department of Orthopedics, The First Affiliated Hospital of Xinxiang Medical University, Xinxiang, Henan, China

**Keywords:** albumin, antibiotic bone cement spacer, C-reactive protein, CRP and albumin ratio, periprosthetic joint infection, prognostic nutritional index

## Abstract

**Introduction:**

This study aimed to evaluate the value of inflammation and nutrition markers in predicting the failure of prosthesis removal and antibiotic bone cement spacer implantation (PRABCSI) for periprosthetic joint infection (PJI) treatment.

**Materials and methods:**

Data from 78 patients with PJI who received PRABCSI were retrospectively analyzed. Patients were divided into a successful group and a failed group according to the outcome at the last follow-up. Patient demographics and laboratory values (white blood cell count, hemoglobin, C-reactive protein [CRP], erythrocyte sedimentation rate [ESR], ESR and CRP ratio [ESR/CRP], lymphocytes, platelet count [PLT], albumin, CRP and albumin ratio [CAR], and prognostic nutritional index [PNI]) were compared.

**Results:**

The successful group had lower levels of CRP, ESR/CRP, PLT, and CAR compared to the failed group. Conversely, the successful group had higher levels of albumin and PNI. The markers with the highest predictive value for PRABCSI failure were CRP and CAR. Elevated levels of CRP and low levels of CAR were associated with a higher risk of PRABCSI failure.

**Conclusions:**

CRP>35.43 and CAR>0.847 are associated with a higher risk of PRABCSI failure in PJI treatment and may serve as preoperative risk-stratification tools.

## Introduction

Despite two-stage revision surgery being selected as the first choice for chronic PJI treatment by most surgeons, its failure rate is around 10% (Barton et al., 2020). As the first step of two-stage revision surgery, the main role of prosthesis removal and antibiotic bone cement spacer implantation (PRABCSI) is to eradicate infections, and its clinical outcome determines whether the second step of two-stage revision surgery can be performed as expected. Although a history of prior revision surgery and the presence of a sinus tract were demonstrated as two independent risk factors for reinfection in PJI patients treated with PRABCSI in Wang et al.’s study (Wang et al., 2022), these two factors are not modifiable. In order to further decrease the failure rate of PRABCSI, we need to identify modifiable markers related to PRABCSI failure and attempt to modify these markers before PRABCSI.

It has been demonstrated that inflammation and nutritional status are associated with postoperative infections in numerous surgeries (Yi et al., 2015; Bala et al., 2020; Hartman et al., 2022), and several inflammation and nutrition markers have been confirmed to have the ability to predict the failure of Debridement, Antibiotic treatment, and Implant Retention (DAIR) (Wouthuyzen-Bakker et al., 2019; Katakam et al., 2020) and two-stage revision surgery in PJI treatment (Borsinger et al., 2022; Hartman et al., 2022). Despite the extensive study of inflammation and nutrition markers such as CRP, albumin, and PNI in the context of PJI treatment and DAIR procedures, the role of these markers in predicting the failure of PRABCSI remains underexplored.

The purpose of this study is to evaluate the value of inflammation and nutrition markers such as white blood cell (WBC) count, hemoglobin (HGB), C-reactive protein (CRP), erythrocyte sedimentation rate (ESR), ESR and CRP ratio (ESR/CRP), lymphocytes (LCT), platelet count (PLT), albumin, CRP and albumin ratio (CAR), and prognostic nutritional index (PNI) in predicting the failure of PRABCSI in PJI treatment.

## Materials and methods

### Patient collective

This retrospective study was approved by the Ethics Committee of our hospital and enrolled patients who received prosthesis removal and antibiotic bone cement spacer implantation (PRABCSI) due to PJI in our center from June 2016 to October 2020. The inclusion criteria were listed as follows: 1) Patients diagnosed with PJI after total hip or knee arthroplasty according to the MSIS (Muscular Skeletal Infection Society) diagnostic criteria (Parvizi et al., 2013); 2) Patients who received prosthesis removal and antibiotic bone cement spacer implantation in our hospital due to PJI; 3) Patients with preoperative data for WBC count, HGB, CRP, ESR, ESR/CRP, LCT, PLT, and albumin. The exclusion criteria were: 1) duration of follow-up less than 2 years; 2) incomplete demographic, clinical, or laboratory data; 3) re-infection not confirmed; 4) interrupted or uncooperative follow-up; 5) combined periprosthetic fracture, dislocation or tumor.

### Surgical process and antibiotic regimen

After successful anesthesia, the skin was incised to expose the joint cavity. Joint fluid and diseased tissue were obtained and sent for etiological examination. If a sinus tract was present, it was removed thoroughly. Inflammatory necrotic tissue and secretion around the prosthesis were debrided thoroughly, the original prosthesis was safely removed, and an antibiotic bone cement spacer was implanted.

The antibiotic regimen in the cement was determined based on the results of preoperative etiological culture. If the preoperative culture was gram-positive, 6 g vancomycin and 2 g imipenem/cilastatin sodium per 80 g package of bone cement were used. If the culture was gram-negative, 2 g vancomycin and 6 g imipenem/cilastatin sodium per 80 g package of bone cement were used. If the preoperative culture was negative or indicated coinfection, 6 g vancomycin and 2 g imipenem/cilastatin sodium per 80 g package of bone cement were used. If the preoperative culture was fungal, 8 g amphotericin per 80 g package of bone cement were used. After the bone cement solidified, fixation was checked, a drainage tube was placed, and the incision was sutured.

Susceptible antibiotics were administered intravenously for 1–2 weeks postoperatively (vancomycin was used if culture was negative) based on susceptibility test results, followed by oral sensitive antibiotics for 4–6 weeks (ciprofloxacin and rifampicin were administered orally if culture was negative). For revision surgery, at least 3 months after PRABCSI, we determined whether the infection was controlled by observing clinical symptoms and serological parameters (blood routine, CRP, and ESR). If infection symptoms resolved and serological indicators decreased to normal levels, a new prosthesis was implanted according to the patient’s preference. Some patients reported satisfactory function with the mobile cement spacer and did not wish to undergo second-stage revision surgery. If serological indicators could not be reduced to normal due to underlying disease, the managing team determined whether the patient could undergo two-stage revision surgery based on clinical symptoms and the downward trend of serological indicators after discontinuing oral antibiotics.

### Definition of success and failure of PRABCSI

Success of PRABCSI was defined as follow-up for at least 2 years without any evidence of reinfection.

Failure of PRABCSI was defined as meeting one of the following three conditions during at least 2 years of follow-up after PRABCSI:

1. failure to achieve effective infection control (evidence of persistent or recurrent infection, as indicated by positive cultures from subsequent samples or clinical signs such as fever, erythema, or purulent discharge), requiring increased antibiotic therapy or reoperation;
2. PJI occurring after the second step of two-stage revision surgery, with the pathogenic bacteria matching those identified during PRABCSI;
3. mixed PJI occurring after the second step of two-stage revision surgery, with the etiological findings containing the pathogenic bacteria detected during PRABCSI.

In this patient group, the presence of reinfection served as the endpoint of observation.

### Justification for including mixed infections in the failure category

MSIS diagnostic criteria: According to the MSIS criteria (Parvizi et al., 2013), mixed infections are considered a form of periprosthetic joint infection (PJI). The presence of multiple pathogens in the infected site indicates a failure to achieve effective infection control, which aligns with our definition of treatment failure. Therefore, we classified mixed infections as failures to reflect the ongoing nature of infection despite treatment.IDSA Guidelines: The IDSA guidelines (Osmon et al., 2013) also support the classification of mixed infections as a significant form of PJI. These guidelines emphasize the complexity and persistence of mixed infections, which often require more aggressive treatment strategies. Given the challenges associated with managing mixed infections, we deemed it appropriate to include them in the failure category to accurately assess the efficacy of the PRABCSI procedure.

### Reinfection types and exclusion criteria

Reinfection types: In our study, we classified reinfections into two categories: prosthetic-related reinfections and hematogenous reinfections. Prosthetic-related reinfections were defined as infections directly related to the implanted prosthesis or the spacer. Hematogenous reinfections were defined as infections resulting from blood-borne pathogens. Exclusion criteria for secondary infections: Secondary infections unrelated to the initial PJI or the PRABCSI procedure were excluded from the study. These included infections occurring due to other surgical procedures, systemic infections not originating from the joint, or infections caused by pathogens unrelated to the initial infection.

### Measuring methods

Preoperative WBC count, HGB, LCT, PLT, albumin, CRP, ESR, ESR/CRP, CAR, and PNI were recorded and compared between the two groups. PNI was calculated as albumin (g/L) +5 * LCT (10^9^/L). The area under the receiver operating characteristic (ROC) curve (AUC), optimal threshold value, sensitivity, and specificity of WBC count, HGB, CRP, ESR, ESR/CRP, LCT, PLT, albumin, CAR, and PNI in predicting PRABCSI failure in PJI treatment were calculated.

### Statistical analysis

Quantitative data were recorded as mean ± standard deviation. An independent-sample t test and chi-square test (χ2) were selected for quantitative and categorical data comparisons between the two groups, respectively. A P value less than 0.05 was considered statistically significant. These statistical analyses were carried out using IBM SPSS Statistics (version 19, IBM SPSS Software).

The performance of CRP, ESR/CRP, PLT, albumin, CAR, and PNI in predicting PRABCSI failure in PJI treatment was evaluated through receiver operating characteristic (ROC) analyses using MedCalc 19.0.4 (MedCalc Software, Ostend, Belgium), assessing parameters such as sensitivity, specificity, and the AUC. Youden’s index was used to define optimal thresholds.

## Results

**Figure 1:**
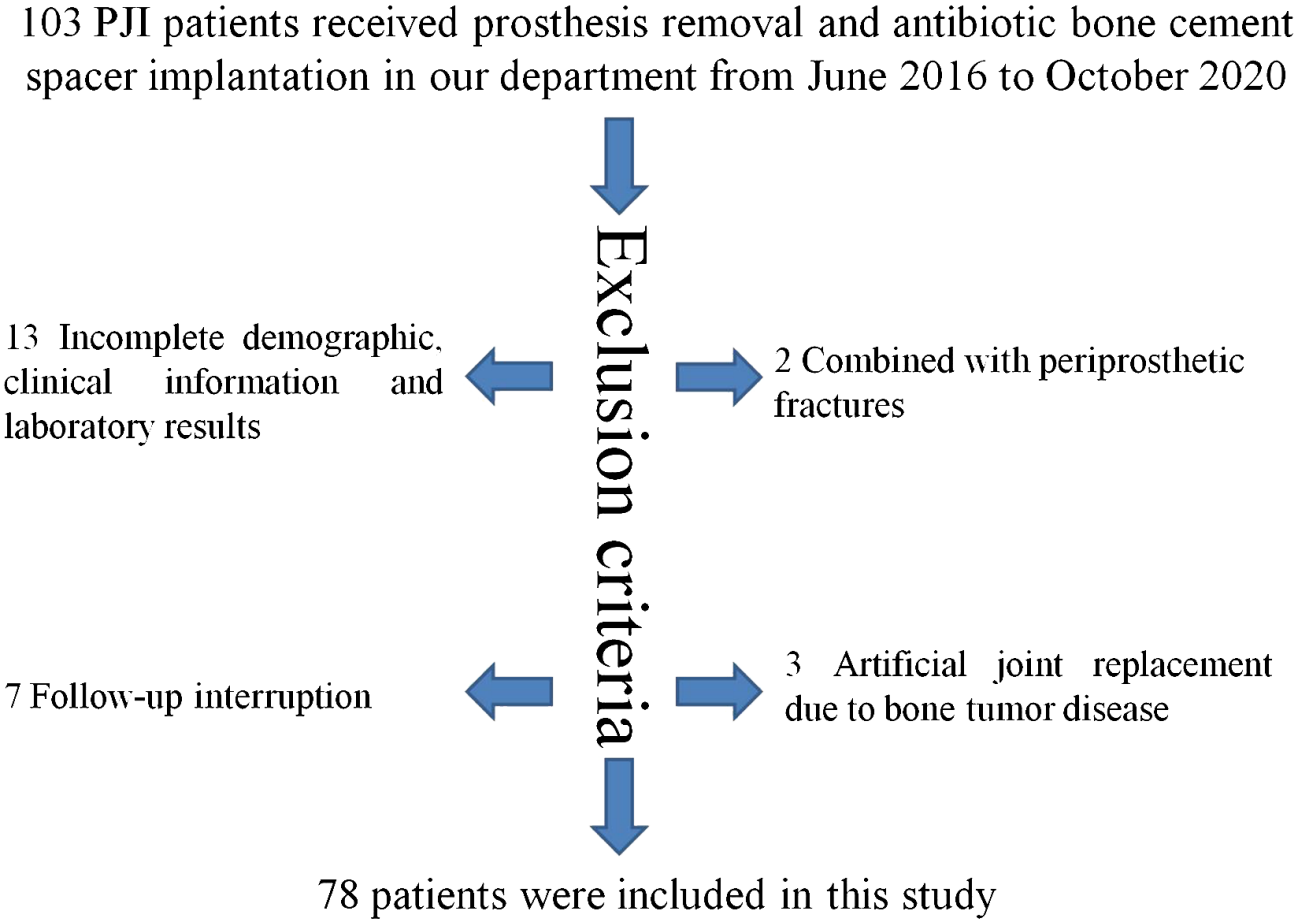
Flow diagram of included patients.

A total of 103 PJI cases were enrolled in this study from June 2016 to October 2020 in our department. Thirteen cases were excluded due to incomplete demographic, clinical, or laboratory data; 7 cases were excluded due to interrupted follow-up; 3 cases were excluded because they had received primary joint arthroplasty for bone tumor disease; and 2 cases were excluded because they were combined with periprosthetic fractures. Finally, 78 patients were included in this study according to the inclusion and exclusion criteria (**Figure 1**).

Patients were divided into two groups (successful group and failed group) according to the clinical outcome after PRABCSI. In the successful group, 56 of the 61 patients were evaluated for serum markers before reimplantation and underwent the second step of two-stage revision surgery after PRABCSI, while the remaining 5 patients did not receive a new prosthesis due to other factors such as patient preference or physical limitations. In the failed group, 14 of the 17 patients were evaluated for serum markers 3 months after PRABCSI; 8 underwent PRABCSI again, 2 underwent debridement, and 2 did not undergo further surgical treatment. The remaining 3 patients did not receive serum or synovial tests after PRABCSI due to persistent sinus tract presence.

Patient demographics are presented in **Table 1**, and no significant differences were found between the two groups when demographic characteristics were compared.

**Table 1:** Demographic characteristics.

Parameters	Success (n=61)	Failed (n=17)	p-value
Sex, n (%)			0.325*
Male	26 (42.62)	5 (29.41)	
Female	35 (57.38)	12 (70.59)	
Joint involved, n (%)			0.642*
Knee	39 (63.93)	9 (52.94)	
Hip	22 (36.07)	8 (47.06)	
Sinus status, n (%)			0.052*
Positive	17 (27.87)	9 (52.94)	
negative	44 (72.13)	8 (47.06)	
HOSBP, n (%)			0.700*
Yes	22 (36.07)	7 (41.18)	
No	39 (63.93)	10 (58.82)	
Comorbidities, n (%)			0.115*
Hypertension	41 (67.21)	12 (70.59)	
Diabetes mellitus	12 (19.67)	6 (35.29)	
Coronary disease	19 (31.15)	4 (23.53)	
Autoimmune diseases	1 (1.64)	3 (17.65)	
Mean age, yrs (SD)	66.57 (11.59)	62.59 (11.24)	0.211†
Mean DOF, months (SD)	39.16 (15.41)	45.29 (19.33)	0.175†
Mean IBISAIPA, months (SD)	34.84 (41.42)	32.13 (45.87)	0.816†
Mean IBUSAP, months (SD)	7.62 (11.12)	8.35 (20.27)	0.845†

*Chi-squared test.

†Independent-samples *t*-test.

DOF, duration of follow up; POI, position of infection; IBISAIPA, interval between the infection symptom and indexed primary arthroplasty; IBUSAP, interval between the infection symptom and PRABCSI; HOSBP, history of surgery before PRABCSI.

**Figure 2:**
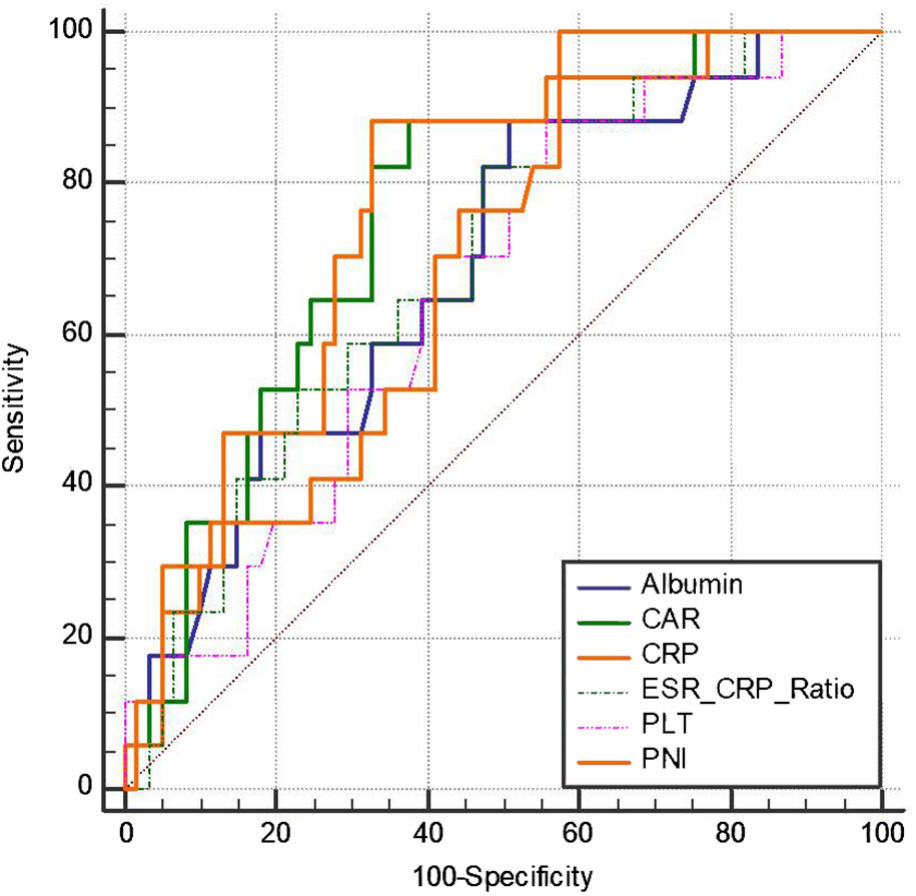
The receiver operating characteristic curve of CRP, ESR/CRP, PLT, Albumin, CAR and PNI.

In order to find out markers that can be used for predicting the failure of PRABCSI in PJI treatment, levels of preoperative WBC count, HGB, LCT, PLT, albumin, CRP, ESR, ESR/CRP, CAR, and PNI were first compared between the two groups. As shown in **Table 2**, the levels of preoperative CRP, PLT, and CAR in the successful group were lower than in the failed group, whereas the levels of preoperative ESR/CRP, albumin, and PNI in the successful group were higher than in the failed group. Secondly, ROC curves and the area under the ROC curve (AUC) were used to define the optimal threshold values and the specificity and sensitivity of these markers in predicting PRABCSI failure in PJI treatment. As shown in **Figure 2** and **Table 3**, CRP exhibited the highest AUC value when used to predict PRABCSI failure (0.765), followed by CAR (0.764), PNI (0.698), ESR/CRP (0.695), albumin (0.689), and PLT (0.660). It appears that CRP and CAR have better performance in predicting PRABCSI failure compared with ESR/CRP, PLT, albumin, and PNI. When 35.43 and 0.847 were set as the optimal threshold values for CRP and CAR in predicting PRABCSI failure, CRP and CAR exhibited specificity values of 67.21% and 62.30%, respectively, and the same sensitivity value of 88.24%. Time-to-event analyses were not performed because the low event number (n = 17) precluded robust survival modeling.

**Table 2:** Comparison of different markers between the two groups.

Parameters	Successful	Failed	p-value
Mean CRP, mg/L (SD)	35.74(36.7)	68.25 ± 45.33	0.003†
Mean ESR, mm/h (SD)	57.20 ± 31.47	71.59 ± 22.47	0.082†
Mean ESR/CRP, (SD)	3.22 ± 3.03	1.59 ± 1.32	0.034†
Mean WBC, × 10^9^/L (SD)	6.91 ± 2.60	7.41 ± 3.76	0.524†
Mean PLT, × 10^9^/L (SD)	285.33 ± 106.85	349.53 ± 121.18	0.037†
Mean HGB, g/L (SD)	114.08 ± 20.9	106.06 ± 14.75	0.143†
Mean LCT, × 10^9^/L (SD)	1.68 ± 0.53	1.52 ± 0.54	0.272†
Mean Albumin, g/L (SD)	35.19 ± 5.25	31.76 ± 5.07	0.019†
Mean CAR, (SD)	1.15 ± 1.40	2.31 ± 1.88	0.007†
Mean PNI, (SD)	43.58 ± 6.16	39.35 ± 5.15	0.012†

†Independent-samples *t*-test.

**Table 3:** The diagnostic performance of different markers in predicting the failure of PRABCSI in PJI treatment.

Markers	AUC	95% confidence interval	Associated criterion	Youden index J	Sensitivity(%)	Specificity(%)	Significance level P (Area=0.5)
CRP	0.765	0.655-0.853	>35.43	0.5545	88.24	67.21	<0.0001
ESR/CRP	0.695	0.581-0.795	≤2.051	0.3481	82.35	52.46	0.0045
PLT	0.660	0.544-0.764	>253	0.3250	88.24	44.26	0.0232
Albumin	0.689	0.574-0.789	≤35.7	0.3742	88.24	49.18	0.0076
CAR	0.764	0.654-0.853	>0.847	0.5053	88.24	62.30	<0.0001
PNI	0.698	0.583-0.797	≤45.7	0.4262	100.00	42.62	0.0024

All these data indicate that CRP>35.43 and CAR>0.847 are associated with higher risk of PRABCSI failure in PJI treatment and may serve as preoperative risk-stratification tools.

## Discussion

Nowadays, two-stage revision surgery is still the first choice by most surgeons for chronic PJI treatment, and its overall success rate is nearly 90% when patients are reviewed and followed for at least 2 years postoperatively (Ma et al., 2018; Petis et al., 2019). Although PRABCSI is the most vital step in two-stage revision surgery and its clinical outcome determines whether the second-stage reimplantation surgery can be performed smoothly, only a few studies have evaluated the rate and risk factors of PRABCSI failure in PJI treatment (Lee and Choi, 2012; Corona et al., 2020; Wang et al., 2022). Despite many studies reporting the significance of blood markers in predicting the outcomes of DAIR and two-stage reimplantation (Sabry et al., 2014; Ma et al., 2018; Wouthuyzen-Bakker et al., 2019; Hartman et al., 2022), it remains unclear whether these markers can predict the failure of PRABCSI in the treatment of PJI. Actively seeking blood markers that can predict PRABCSI failure and adjusting these abnormal indicators preoperatively may provide new insights for improving the clinical efficacy of PJI treatment. In this study, our results showed that CRP > 35.43 and CAR > 0.847 are associated with a higher risk of PRABCSI failure in PJI treatment and may serve as preoperative risk-stratification tools. To our knowledge, this is the first study to evaluate the role of CRP, ESR/CRP, PLT, albumin, CAR, and PNI in predicting PRABCSI failure.

Although CRP and ESR are the most commonly used blood markers for the diagnosis of infectious diseases, they also have been demonstrated to predict the failure of two-stage reimplantation (Sabry et al., 2014; Hartman et al., 2022). In this study, our data also showed that the level of CRP in the failed group is significantly higher than in the successful group, but the difference in ESR levels between the two groups was not significant. The underlying reason may be that CRP is a marker of acute inflammation (Pepys and Hirschfield, 2003), whereas ESR is a marker of chronic inflammation (Lapic et al., 2020). Different from the patients included in studies published by Fady et al. Sabry et al. (2014) and Curtis et al (Hartman et al., 2022), all the included patients in our study were diagnosed with chronic PJI, and there were no differences in the interval between infection symptoms and the indexed primary arthroplasty or the interval between infection symptoms and PRABCSI. Therefore, the ESR level between the two groups should be similar. However, regarding the difference in CRP levels, we think higher CRP represents a relatively “acute” status, as increased CRP indicates that the immune system is still struggling to control the infection or is under assault by a more virulent pathogen (Hartman et al., 2022). In order to further validate this hypothesis, ESR/CRP—which has the ability to distinguish acute and chronic inflammatory status (Christopher et al., 2021), was compared between the groups. As expected, ESR/CRP in the failed group was lower than in the successful group. However, only the AUC of CRP in predicting PRABCSI failure was > 0.7, suggesting that CRP could predict PRABCSI failure.

As a high CRP level indicates either that the pathogen is highly virulent or that the immune system is relatively weak. (Hartman et al., 2022), and because Staphylococcus species infection confers a higher risk of failure for DAIR) (Tarity et al., 2021) and two-stage revision surgery (Hartman et al., 2022), the rate of Staphylococcus species infection between the two groups was compared. In this study, there was no difference in the rate of Staphylococcus species infection between the failed group (23.50%, 4/17) and the successful group 19.70% (12/61) was compared (χ2 = 0.000, p=0.993) (**Supplementary Table S1**). This indicated that the immune system in the failed group may be weaker than in the successful group.

Malnutrition is one of the main causes of a weak immune system and is associated with postoperative infections in various surgical procedures. It has been reported that nearly 50% of orthopedic patients suffer from malnutrition before surgery (Pruzansky et al., 2014; Golladay et al., 2016), so we assessed whether malnutrition-evaluating markers (PLT, WBC count, LCT, HGB, albumin, and PNI) (Kurtz et al., 2018; Maimaiti et al., 2021) can predict the failure of PRABCSI in PJI treatment. As expected, the levels of albumin and PNI in the failed group were significantly lower than in the successful group. However, both the AUC of albumin and PNI in predicting PRABCSI failure were < 0.7. Moreover, there were no significant differences when levels of WBC count, LCT, and HGB were compared between the failed and successful groups. We think these results may be related to the small sample size, and because WBC count, LCT, and HGB can be affected by many factors other than malnutrition. Additionally, different from expectation, our data showed that the level of PLT in the failed group was much higher than in the successful group, and the AUC of PLT in predicting PRABCSI failure was < 0.7. We think this result may be related to the fact that PLT level is easily affected by various factors (infection status, coagulation status), and both abnormally high and low preoperative PLT correlate with adverse outcomes after elective total knee arthroplasty (Malpani et al., 2019). All these data indicate that PLT, WBC count, LCT, HGB, albumin, and PNI do not perform well in predicting PRABCSI failure.

Recently, CAR was proven to be not only a better diagnostic marker for PJI than CRP and ESR (Shi et al., 2021), but also a useful biomarker for predicting surgical site infection after major abdominal surgery (Donlon et al., 2020). In this study, we also found that CAR in the failed group was higher than in the successful group, and the AUC of CAR in predicting the failure of PRABCSI in PJI treatment was 0.764, which implied that CAR is associated with a higher risk of failure.

Different from the previously published failure rates of PRABCSI in PJI treatment reported by Corona et al. (11.11%) (Corona et al., 2020) and Lee et al. (10.53%) (Lee and Choi, 2012), the overall failure rate in our study was 21.79% (17/78). The underlying reason for the discrepant failure rate may be related to the different definitions of PRABCSI failure. In the studies by Corona et al. and Lee et al., they defined failure only as patients who still had typical infection symptoms after PRABCSI. However, patients who still had persistent infection without typical symptoms after PRABCSI were missed from the failure group. These patients may suffer PJI after the second step of two-stage revision surgery caused by the same pathogenic bacteria detected during PRABCSI. As a result, the failure rate in our study is much higher than that reported by Corona et al. and Lee et al., but we think that our result is more reasonable. Furthermore, the overall failure rate of PRABCSI in our study (21.79%) is also significantly higher than the failure rate (10%) of two-stage revision surgery in PJI treatment (Masri et al., 2007; Ma et al., 2018; Petis et al., 2019). This phenomenon indicates that markers used to predict failure of two-stage revision surgery in PJI treatment (Ma et al., 2018; Borsinger et al., 2022) may not be applicable for predicting PRABCSI failure. Therefore, it is necessary to identify markers suitable for predicting PRABCSI failure in PJI treatment.

In this study, we firstly evaluated the value of several daily used blood markers such as CRP, ESR/CRP, PLT, albumin, CAR, and PNI in predicting the failure of PRABCSI in PJI treatment, and demonstrated that, compared with ESR/CRP, PLT, albumin, and PNI, CRP and CAR can predict the failure of PRABCSI in PJI treatment. We think this finding has a profound impact on PJI management: 1) for those who presented with CRP > 35.43 and CAR > 0.847 before PRABCSI, more attention should be paid to evaluate whether the infection was controlled after PRABCSI. Synovial fluid aspiration or histologic analysis of synovial tissues should be performed before the implementation of two-stage reimplantation surgery to evaluate whether the infection was controlled or not. For those with suspiciously uncontrolled infection, the implementation of two-stage reimplantation surgery should be delayed; 2) for those who presented with modifiable markers such as albumin ≤ 35.7 and PNI ≤ 45.7 before PRABCSI, diet or drug therapy should be applied to modify these markers to acceptable levels, and whether correction of hypoalbuminemia or improvement of PNI by nutritional support can translate into better outcomes should be tested in prospective trials.

Notwithstanding, there are several limitations in our study: 1) this was a retrospective, single-center study, which may have selection bias; 2) our study included a total of 78 patients, with 17 failure events. Given the modest sample size and the low number of failure events, the statistical power to detect smaller differences or to support extensive multivariable modeling is limited. Despite these limitations, several markers reached statistical significance, which is noteworthy. However, this also raises the risk of type I error (false positives) due to multiple comparisons. We acknowledge that the limited sample size could potentially affect the robustness of our findings and the generalizability of our conclusions. Future studies with larger sample sizes and more failure events are needed to validate our findings and to provide more robust statistical analyses. 3) In this study, we included 78 out of 103 PJI cases, with the exclusion of cases primarily due to incomplete demographic, clinical information, and laboratory results. The exclusion of cases with incomplete data was necessary to ensure the accuracy and reliability of our analyses. However, this decision may have inadvertently led to a non-representative sample. For instance, cases with incomplete data might have different characteristics compared to those with complete data. Specifically, patients with incomplete records could have more severe or less severe conditions, leading to an overestimation or underestimation of the prevalence and outcomes of PJI. So, we recommend that future research employs more robust methods to handle missing data, such as multiple imputation techniques, to provide a more accurate representation of the population.

4) this study had a large time span, and PRABCSI procedures were not performed by the same surgeon, which could not remove the differences in results caused by different surgeons;

5) the number of included patients in our study was only 78, and the AUC values of ESR/CRP (0.695), PLT (0.660), albumin (0.689), and PNI (0.698) in predicting failure of PRABCSI in PJI treatment were slightly lower than 0.7. Larger and higher-quality studies are still needed to evaluate the value of these markers in predicting failure of PRABCSI; 6) we acknowledge the potential for temporal bias due to differences in follow-up duration. We believe that our current approach, which includes a detailed comparison of patient demographics and outcomes, already provides a robust assessment of treatment efficacy. The difference in mean follow-up duration between the groups (39.16 months *vs*. 45.29 months) was not statistically significant (p=0.175), suggesting that temporal bias is not a significant concern in our study. Given the exploratory nature of our study and the relatively small sample size, we chose to focus on a simpler and more clinically relevant approach;

7) we acknowledge that the inclusion of both hip and knee PJI cases in a single analysis could be a limitation if not properly addressed. Our data indicate that there were no significant differences in the proportions of hip and knee cases between the success and failure groups. This suggests that the overall findings are not significantly influenced by the type of joint involved. However, we recognize that larger, more focused studies on hip and knee PJI cases separately would be beneficial to further validate these findings; 8) while multivariable analysis can provide insights into the independent predictive value of markers, we believe that our current approach—which includes ROC analysis and the evaluation of multiple markers—already provides a comprehensive assessment of the predictive value of CRP and CAR in the context of PRABCSI. Given the exploratory nature of our study and the relatively small sample size, we chose to focus on a simpler and more clinically relevant approach. The differences in potential confounders such as sinus tract and autoimmune diseases between the groups were not statistically significant, suggesting that their impact on the primary outcome may be minimal;

9) because only 17 failure events were observed, ROC-derived thresholds should be regarded as exploratory and potentially unstable. Bootstrapping or other resampling techniques could not be applied meaningfully in this small-event setting. Additionally, multivariable logistic or Cox regression was not feasible. All comparisons are therefore univariate and potentially confounded by factors such as diabetes, sinus tract formation, pathogen virulence, inflammatory burden, or prior revision surgery. Consequently, the reported cut-off values require external validation before any clinical application; 10) we selected the Youden Index to determine the optimal threshold for evaluating the value of these markers in predicting failure of PRABCSI. However, we recognize that the Youden Index has certain limitations, such as assuming equal costs for false positives and false negatives, whereas in some clinical scenarios these costs may differ. Additionally, the Youden Index does not account for absolute error probabilities, focusing solely on maximizing the combination of sensitivity and specificity. Future research could explore alternative methods (such as cross-validation, cost-effectiveness analysis, and Bayesian methods) and validate them across larger sample sizes and diverse clinical settings to identify more suitable threshold-selection approaches, thereby enhancing the clinical utility of these blood markers in predicting failure of PRABCSI.

## Conclusion

In conclusion, the present study first showed that CRP > 35.43 and CAR > 0.847 are associated with a higher risk of failure of PRABCSI in PJI treatment and may serve as preoperative risk-stratification tools. More actions should be taken to decrease the failure rate of PRABCSI in PJI treatment when patients present with these markers.

## Data Availability

The raw data supporting the conclusions of this article will be made available by the authors, without undue reservation.

## Supplementary material

Supplementary Table 1 Type of pathogens in the two groups.
