# Supplementary material for: Value of inflammation and nutrition markers in predicting the failure of prosthesis removal and antibiotic bone cement spacer implantation for PJI treatment
Source: Front Cell Infect Microbiol. 2025 Dec 19;15:1610156. doi: 10.3389/fcimb.2025.1610156 (PMC12757415; doi:10.3389/fcimb.2025.1610156)
Supplement: Supplementary Table 1 — Type of pathogens in the two groups. [file Table1.docx]

| **Supplementary Table S1 Type of pathogens in the two groups** | | | | |
| --- | --- | --- | --- | --- |
| **Pathogens** | Successful | | Failed | |
| *Negative culture* | | 16 | | 4 |
| *Staphylococcus haemolyticus* | | 2 | | 0 |
| *Staphylococcus aureus* | | 11 | | 4 |
| *Staphylococcus epidermidis* | | 8 | | 0 |
| *fungus* | | 4 | | 1 |
| *Staphylococcus haemolyticus* | | 2 | | 0 |
| *Escherichia coli* | | 2 | | 2 |
| *mixed infection* | | 4 | | 1 |
|  | | | | |
